# Low-quality protein modulates inflammatory markers and the response to lipopolysaccharide insult: the case of lysine

**DOI:** 10.1017/S0007114522004068

**Published:** 2023-09-28

**Authors:** Carla El-Mallah, Marie-Elizabeth E. Ragi, Assaad Eid, Omar A. Obeid

**Affiliations:** 1 Department of Nutrition and Food Science, Faculty of Agricultural and Food Sciences, American University of Beirut, Beirut, Lebanon; 2 Department of Anatomy, Cell Biology, and Physiological Sciences, Faculty of Medicine, American University of Beirut, Beirut, Lebanon

**Keywords:** Lysine, Deficiency, Albumin, C-reactive protein, Anxiety-like behaviour, Rats

## Abstract

The relationship between non-communicable diseases and eating behaviour has long been attributed to a surplus of food and energy. However, the increase in the prevalence of non-communicable disease and their underlying low-grade inflammatory milieu among people of low socio-economic status has highlighted the existence of a confounding factor. In this work, we aim to study the effect of lysine deficiency on some inflammatory markers in the absence or presence of an inflammatory insult (lipopolysaccharide (LPS)). For this purpose, thirty-two 5-week-old male Sprague Dawley rats were randomly distributed into four groups: (1) control diet, (2) control diet+LPS, (3) lysine-deficient diet and (4) lysine-deficient diet + LPS. Groups were only allowed their experimental diets for 4 weeks, during which LPS (50 µg/kg) or saline injections were administered intraperitoneally three times per week. The study showed that lysine deficiency blunted growth and body compartments development, decreased albumin production and elevated liver C-reactive protein (CRP) expression, independently of IL-6 and IL-1*β*, the main precursors of CRP. Also, the insufficient levels of lysine in the diet increased hyperactivity and triggered an anxiety-like behaviour, exacerbated with LPS. This work presents evidence that various physiological changes are associated with the absence of a sufficient amount of lysine in the diet and can potentially increase the risk factor for diseases. Thus, the increment in non-communicable disease among the low socio-economic status populations, who heavily rely on cereals as a main source of protein, can be, at least partially, blamed on low lysine availability in diets.

Over the last decades, the world has witnessed an increase in non-communicable diseases among low- and middle-income countries^(1)^. This comes along with many reports showing higher levels of inflammatory markers, notably C-reactive protein (CRP), among subpopulations with low socio-economic status^(2–4)^, affirming the dogma that low-grade inflammation plays a pivotal role in the pathophysiology of many diseases^(5)^.

The increased prevalence of non-communicable diseases and their attributable deaths can be, at least partially, explained by some dietary factors associated with the nutrition westernisation phase^(6)^. This dietary pattern is characterised by high fat, refined carbohydrates and processed foods^(7)^. Due to the high cost of animal proteins, low socio-economic status communities have adopted the westernised pattern but kept their primary protein intake from plant sources^(7)^, mainly cereals, which sometimes reached more than 80 % of the daily protein intake^(8)^.

Cereals (rice, wheat and maize) significantly contribute to the total energy intake of many populations; nonetheless, their proteins do not contain a sufficient amount of lysine^(9)^, an essential amino acid that cannot be produced by the body and needs to be procured from the diet^(10)^. This makes lysine the first limiting amino acid in cereals^(10)^. For instance, wheat shares the lowest lysine content of 22–35 mg/g of protein, followed by rice (30–36 mg/g) and maize (28–42 mg/g)^(11)^, while animal products such as meat, poultry, fish and dairy products have high lysine content, more than 2·7-fold the amount found in plant-based products^(8)^. When the digestible indispensable amino acid score (DIAAS) was evaluated in food items, the lowest DIAAS range was attributed to cereals (1 % to 77 %), with lysine as the limiting indispensable amino acid, followed by legumes and nuts (43 % to 105 % and 83 % to 86 %, respectively), and animal-based products (ranging from 80 % and reaching 144 %)^(12)^.

In animals, adding 5 % casein (animal-based protein) to the wheat gluten diet was found to improve the quality of protein and prevent the decline in weight gain, food intake and feed efficiency^(13)^. On the other hand, exclusively consuming incomplete gluten proteins, Sprague Dawley rats had defects in growth, development and energy efficiency^(14)^.

Several lysine fortification or supplementation trials were shown to produce paramount effects at different levels. When the impact of the fortification of wheat flour with lysine was assessed in Northern China, Zhao *et al.* demonstrated a significant increment in plasma levels of prealbumin, IgG, IgA, IgM and complement factor 3 (C3)^(15)^, which reflects a better nutritional and immunological status. Similar results were also shown in other settings^(16–18)^. Additionally, in a study on rats, lysine supplementation of gluten diets significantly increased weight gain and growth and improved plasma albumin levels^(14)^. Moreover, lysine was also shown to have significant roles at the central levels in modulating anxiety. A 3-month randomised double-blind lysine fortification of wheat reported reduced chronic anxiety among some Syrian communities^(19)^. Also, oral lysine, along with arginine, reduced traits of anxiety and salivary levels of cortisol and chromogranin-A among healthy Japanese adults^(20)^.

The relationship between lysine and general health outcomes displays a potential role of lysine deficiency, among low socio-economic status communities that rely on cereals, in increasing risks of developing non-communicable diseases. We hypothesise that the lack of a sufficient amount of lysine in a diet mediates low-grade inflammation in the absence of infectious stimuli; and that the inflammatory profile in a triggered immune reaction would be altered in the case of lysine deficiency. To our knowledge, this is the first study that aims at studying the effect of lysine deficiency on some inflammatory markers in the context of inflammatory insult induced by lipopolysaccharide (LPS) in growing rats. With a similar nutrient metabolism and inflammatory responses to humans, young male Sprague Dawley rats were used in this study and closely monitored for their growth, metabolisms and behaviour.

## Materials and methods

### Ethical approval

The protocol of this experiment was submitted and approved by the Institutional Animal Care and Use Committee (IACUC) of the American University of Beirut (AUB) (approval # 20–08–577). The study was performed by following the criteria outlined in the Guide for the Care and Use of Laboratory Animals^(21)^. The animals’ health and welfare were checked weekly by the veterinarian of the animal care facility.

### Animal model

Five-week-old male Sprague Dawley rats (*n* = 32), weighing about 170 g, were housed individually in wire-bottom cages in a controlled temperature (22 ± 1°C) and humidity (65 %) room with a light–dark cycle of 12/12 (lights start at 07.00). The required number of rats per group (*n* = 8) was calculated using previously determined weight gain data (difference in means = 6·0 g/d and sd = 0·95), assuming a statistical power of 80 %, a significance level of 5 % and 1·5 difference in means^(22)^.

### Diets and experimental groups

Rats were acclimatised to the room, single caging and the texture of the semi-synthetic powered diet for 3 d. Following an adaptation period, rats were randomly divided into four groups of eight each, as shown below. Different treatments and experimental diets were given according to the group to which they were assigned. They had ad libitum access to water and their corresponding diets over 4 weeks. The diets’ compositions and nutrition facts are presented in Supplementary Tables S1 and S2 of the supplementary material. All following experiments were conducted in random order of rats irrespective of the groups to minimise the time and sequence errors.

Group 1- Control: Control diet providing an adequate amount of lysine (*n*=8).

Group 2- LPS: Control diet providing an adequate amount of lysine + LPS injection (*n* = 8).

Group 3- Lysine-deficient 60 %: Lysine-deficient diet providing about 60 % less lysine than the control diet (*n* = 8).

Group 4- Lysine-deficient 60 %-LPS: Lysine-deficient diet providing about 60 % less lysine than the control diet + LPS injection (*n* = 8).

Gluten-based diets were chosen for this experiment, since gluten (the wheat protein) is naturally deficient in lysine. The control diet is supplemented to meet the lysine requirements in growing rats, according to the National Research Council^(23)^. The diets were designed to be isoenergetic (about 16·42 kJ/g) and isonitrogenous (about 15·4 % of energy from protein).

### Lipopolysaccharide challenge

LPS derived from *Salmonella Typhosa* was purchased from Sigma Aldrich (St. Louis, MO, USA), solubilised in 0·9 % sterile pyrogen-free saline solution for a final concentration of 20 μg/ml and stored at −80°C until use. Before administration, LPS solutions were brought to room temperature and injected intraperitoneally at a dose of 50 μg/kg, three times a week, which sums up to 150 μg/kg per week. The dose of administration was set based on data from Elgarf *et al.* (2014)^(24)^ that showed that repeated exposure (six injections, every other day) of rats to 50 µg/kg over 2 weeks was the lowest effective dose that had significantly produced behavioural changes. Unchallenged groups received a similar volume-to-weight intraperitoneal injection of 0·9 % saline solution. The dose was expected to affect the rats’ behaviour, body composition and immune status, while the frequency of injection would be in line with the 1·9 d of the half-life of the plasma albumin in rats^(25)^.

### Food intake and body composition

Food intake was measured twice per week during the experimental period, and energy intake was then calculated by multiplying the weight of food consumed by its energy content (online Supplementary Table S2). Total energy expenditure (TEE_xp_) was estimated based on the equation by Ravussin *et al.* ^(26)^, and energy efficiency was determined as the amount of weight gained per 1 MJ consumed. Also, body weight and composition were assessed weekly using TD-NMR Minispec LF110 (Brucker), which provides a non-invasive body compartment (body fat, lean mass and free fluids) analysis in living animals based on a magnetic resonance system.

### Blood and tissue collection

By the end of the fourth week, 24 h after the last LPS injection, the rats were euthanised by severing their hearts after a 5- to 6-h fast. The procedure was performed under isoflurane (Forane®; Abbott). Blood was collected in EDTA tubes from superior vena cava, and blood samples were centrifuged at 3500 rpm for 15 min at 4°C (EPPENDORF 5810R). The spleen, epididymal fat and liver were immediately excised and weighed, snap-frozen in liquid N_2_ and then stored at −80°C.

### Fasting plasma analysis

Plasma glucose (Glu), total cholesterol (TC), HDL-cholesterol, TAG, albumin, lactate, Na and chloride were determined using Vitros® 350 Chemistry System (Ortho-Clinical Diagnostics). LDL-cholesterol was calculated using the Friedewald equation: LDL = TC – HDL – TG/5^(27)^. Plasma insulin (Ins) (Merck Millipore) and total glucagon-like peptide-1 (Merck Millipore) were measured using ELISA kits. Homeostatic Model Assessment for Insulin Resistance (HOMA-IR) index was computed using the equation by Antunes *et al.* HOMA IR = Ins (mmol/l) × Glu (mmol/l)/22·5^(28)^.

### Free amino acid determination

The concentrations of plasma free amino acid were quantified by HPLC. In brief, plasma samples (20 μl) were mixed with an equal volume of internal standard (L-homoserine, Sigma Aldrich), then precipitated with 6-sulphosalicylic acid and centrifuged at 15 000 × ***g*** for 10 min at 4°C. The supernatant was filtered, and 10 μl was injected into the HPLC system. The derivatised amino acids by o-phthalaldehyde were separated on a Hichrom Spherisorb ODS reverse phase column (150 × 4·6 mm; Hichrom Ltd). The flow rate was 1·2 ml/min. Twenty amino acids were identified and quantified by dividing the peak area for each amino acid by the one of the internal standard.

### Hepatic fat extraction

Frozen liver sections (about 3 g) were freeze-dried (Benchtop Freeze-Dry System, Labconco) for 48 h. Hepatic fat content was then determined using an ANKOM^XT10^ extractor (ANKOM Technology 2052 O’Neil Road) with petroleum ether (BP 40–60°C) for a cycle of 40 min. After extraction and cooling, samples were put for 40 min at 100°C to dry and then cooled at room temperature in a desiccator. The difference in weight refers to the extracted fat from liver samples.

### Histological analysis

Frozen livers and epididymal adipose tissues were sectioned and fixed in 10 % buffered formalin solution over 24 h, dehydrated in sequential ethanol solutions and embedded in paraffin before being sectioned and mounted on microscope slides. After complete dryness, the slides underwent deparaffinisation, rehydration to distilled water, and staining with hematoxylin and eosin (H&E). Areas of adipocytes were measured using ImageJ software, and quantifications were performed manually for a set area in all slides. A pathologist blinded to the experimental groups assessed the morphology of liver sections.

### Intraperitoneal glucose tolerance test

Room temperature, pH-adjusted 50 % glucose (Sigma Aldrich) solution in 0·9 % saline was administered intraperitoneally to four rats per group at a volume corresponding to 2 g of glucose per kilogram of weight after a 5-h fast. A tail prick with a small needle was done to collect blood drops from the lateral tail vein. The glycemic response was measured with Accu-Check® Guide over 2 h at different time points (baseline before intraperitoneal injections and 15, 30, 90 and 120 min after intraperitoneal injections). The AUC was calculated using Prism, GraphPad software.

### Western blot analysis

Liver tissues were separately homogenised with radioimmunoprecipitation assay lysis buffer containing phosphatase and protease inhibitors (Bioworld). Homogenates were quantified using the DC Protein Assay by Bio-Rad, loaded and separated by electrophoresis. Acrylamide gels were transferred onto a nitrocellulose membrane using the semi-dry transfer method (Bio-Rad). Membranes were blocked in 1 % gelatin/TBS-Tween 0·1 % for 1 h at 37°C and incubated overnight at 4°C with one of the following primary rabbit anti-rat antibodies: anti-*β*-actin (Bio-Rad) and anti-CRP (MyBioSource). Subsequently, and after extensive washing, membranes were incubated for 1 h at room temperature with goat anti-rabbit IgG HRP-conjugated secondary antibody (Bio-Rad). Immunoreactive bands were visualised with enhanced chemiluminescence detection solutions (Bio-Rad, Clarity™ Western ECL Blotting Substrates) using ChemiDoc MP Imaging System (Bio-Rad). The intensity of the bands was quantified with ImageJ and normalised over the corresponding loading reference band (*β*-actin).

### RNA extraction and quantitative RT-PCR

Total RNA was isolated from frozen tissues with Trizol (TRI Reagent®, Sigma-Aldrich). The concentration and purity of the RNA were assessed using the DeNovix DS-11 Series Spectrophotometer (Thermo Fisher Scientific). Satisfactory samples were transcribed into complementary DNA using RT Master Mix for quantitative PCR (qPCR) II (gDNA digester plus) (MedChemExpress) according to the manufacturer’s protocol. The mRNA expression was analysed by qRT-PCR (Bio-Rad CFX^TM^ Manager Software) using a SYBR green master mix (Applied Biosystems). The list of primers used and their sequences are listed in the Supplementary Table S3. The following temperature protocol was used: 95°C for 5 min (for denaturation) and 40 cycles of 95°C for 10 s (s) and 57°C for 30 s followed by 72°C for 30 s. Reactions were performed in duplicates, and expression levels of the target genes were calculated according to the ΔΔCt method using the tyrosine 3-monooxygenase/tryptophan 5-monooxygenase activation protein zeta (WYHAZ) gene as an endogenous control for normalisation. Changes in gene expression were presented relative to the control group.

### Hargreaves test (thermal pain test)

A thermal sensitivity test was undertaken to assess peripheral pain sensation and thus explain discrepancies (if any) in the movement of the animals. The test was performed on all rats a week before euthanasia and 24 h after LPS injection, as follows. Rats were put for 30 min in a transparent glass-floored chamber at a temperature of 32°C for acclimatisation. When rats were still and standing on the four paws, infrared heat was projected from a 160-watt light bulb underneath the chamber to the planter surface of the rat’s hind paw. The withdrawal latency, the time required to elicit a withdrawal response after heat exposure, was recorded in seconds. A cut-off latency of 18 s was used to avoid any tissue damage. This heat stimulus was applied thrice per rat, separated by an interval of 5 min. The average of the two closest values was reported.

### Open-field test

An open-field test was performed on all rats to assess their locomotricity, anxiety, as well as exploratory and hyperactive behaviours. This test took place on three consecutive days during the last week before euthanasia. All animals were accustomed to the experimental room for 30 min before the beginning of the test. Every rat was placed in an enclosure, square (W: 80 cm, L: 80 cm, H: 40 cm), open-lit and surrounded-wall apparatus, enlightened in the centre and dark by the periphery. The rats’ behaviours and trajectories were recorded for 5 min with a video camera connected to the tracking software Smart 3.0 software (PanLab Harvard Apparatus). The primary outcomes discussed in this work are the total distance travelled that identifies hyperactivity^(29)^, the distance travelled in the periphery and the time spent in the corners that show anxiety-like behaviours^(30)^.

### Statistical analysis

Data are presented as mean ± sd or sem. Statistical analysis was performed using IBM Statistical Package for the Social Sciences 25 (IBM SPSS) software. Values were considered outliers and excluded from analysis when they surpassed the mean ± 2 sd, except for the open-field test, where all values were kept. Results were analysed by one-way ANOVA to detect the difference among groups, followed by Fisher’s least significant difference for *post hoc* multiple comparisons. General linear model was also used to assess the effect of the different groups (Groups) over time (Time) or lysine deficiency (Lysine) and the administration of LPS (LPS) (as fixed factors) and their corresponding interaction. Statistical significance was set at a *P*-value of 0·05.

## Results

### Body measurements and energy parameters

**Fig. 1.**
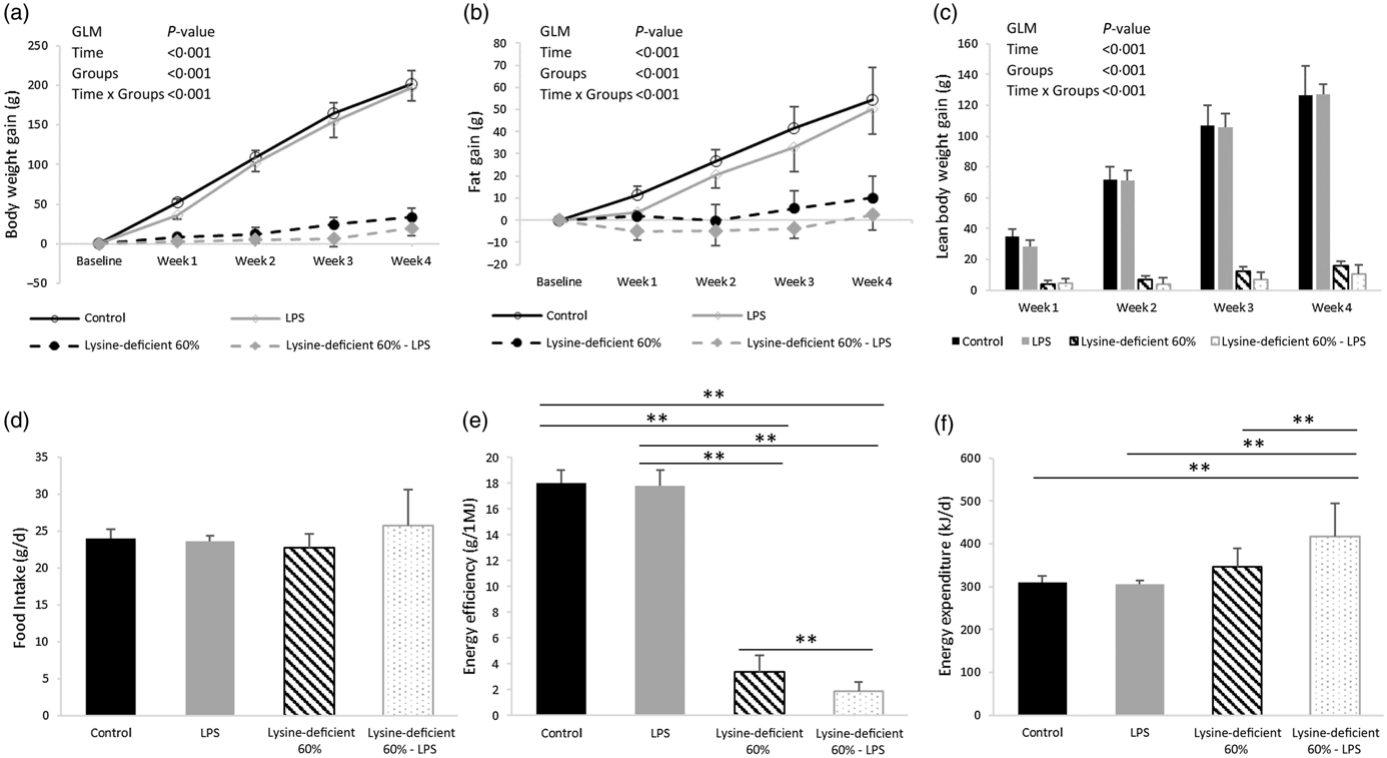
(a) Body weight gain from baseline for 4 weeks, (b) fat gain from baseline for 4 weeks, (c) lean body weight gain from baseline for 4 weeks, and (d) food intake, (e) energy efficiency, and (f) energy expenditure per d of rats fed a control diet or a 60 % lysine-deficient diet in the presence or absence of LPS challenge (*n* = 8 per group). Data are expressed as the mean ± sd and analysed by one-way ANOVA to detect the difference between groups, followed by Fisher’s least significant difference for *post hoc* multiple comparisons. General linear model was used to analyse the effect of different parameters across the experimental period, considering the two fixed factors (Time and Groups) and their interaction (Time × Groups). Statistical significance was set at *P*-value < 0·05. * refers to *P*-value < 0·05. ** refers to *P*-value < 0·01. GLM, general linear model; LPS, lipopolysaccharide.

Body weight changed differently in the presence or absence of a sufficient amount of lysine (control *v*. lysine-deficient diet) (Fig. 1(a)). Despite the little clinical relevance of the small effect shown with LPS administration, the decrease in body weight was statistically significant. The pattern of fat accumulation over the weeks was similar to the one of weight gain (Fig. 1(b)). Nevertheless, the fat percentage was comparable among the groups at baseline and the similarity remained 4 weeks after (*P*-value > 0·05) (online Supplementary Fig. S1). Moreover, the control diet induced a gradual and continuous increase in lean body mass (*P*-value < 0·001), which was blunted with lysine deficiency (Fig. 1(c)). No effect was observed for the LPS on lean body weight (*P*-value = 0·093). While food intake seems constant among the groups (*P*-value = 0·249) (Fig. 1(d)), energy efficiency dropped significantly with lysine deficiency, and it was further reduced in the presence of LPS (Fig. 1(e)). In parallel, energy expenditure was increased in the lysine-deficient 60 %-LPS group (Fig. 1(f)).

**Fig. 2.**
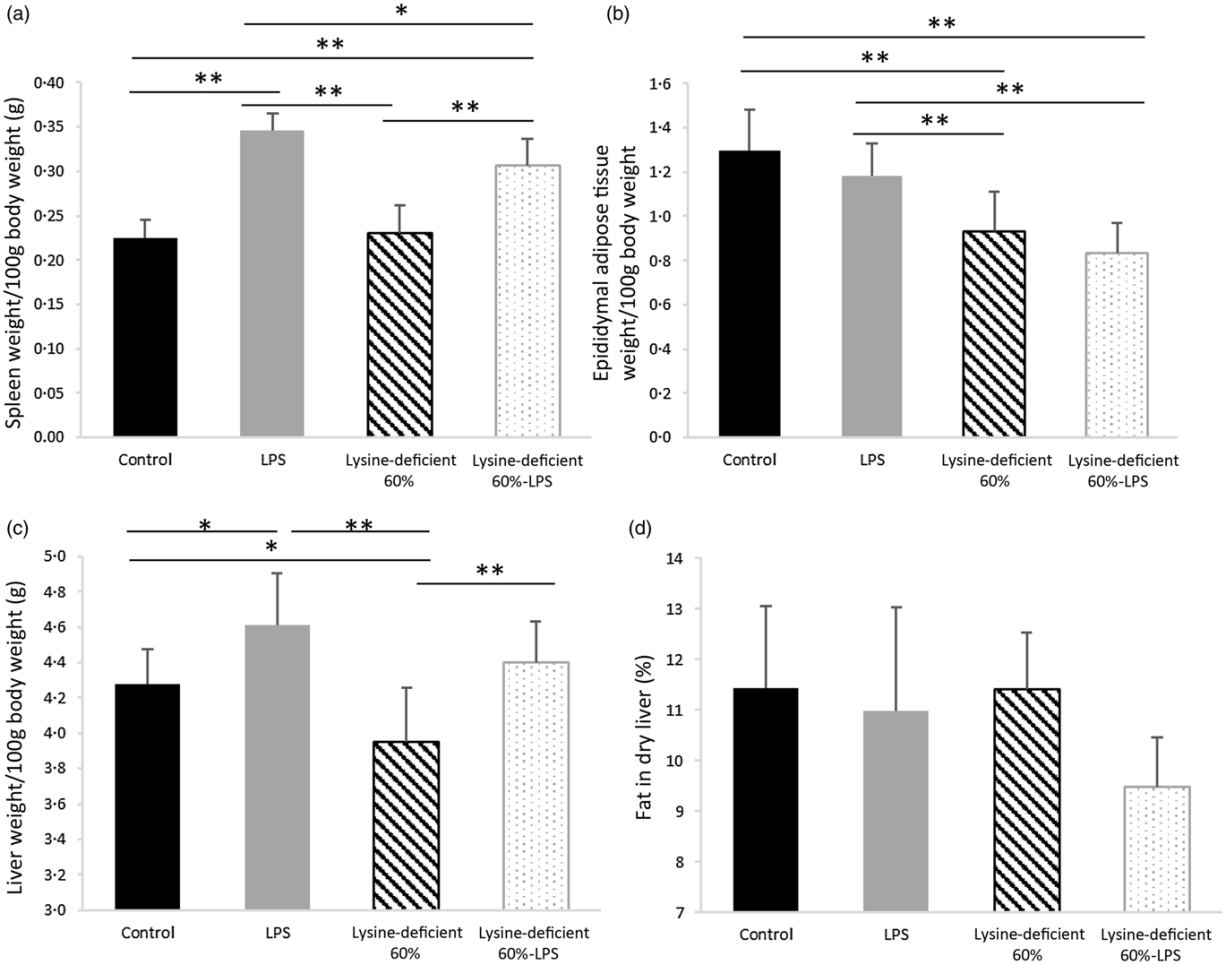
(a) Spleen, (b) epidydimal adipose tissue and (c) liver weights per 100 g of body weight, and (d) percentage of fat content in dry liver of rats fed a control diet or a 60 % lysine-deficient diet in the presence or absence of LPS challenge (*n* = 8 per group). Data are presented as mean ± sd and analysed by one-way ANOVA to detect the difference between groups, followed by Fisher’s least significant difference for *post hoc* multiple comparisons. Statistical significance was set at *P*-value < 0·05. * refers to *P*-value < 0·05. ** refers to *P*-value < 0·01. LPS, lipopolysaccharide.

Organs were excised, weighed and adjusted to the total body weights of rats on the day of the euthanasia. As shown in Fig. 2, weights of the spleen (*P*-value < 0·001) (Fig. 2(a)), epididymal adipose tissue (*P*-value < 0·001) (Fig. 2(b)) and liver (*P*-value = 0·001) (Fig. 2(c)) were different among the groups. LPS (*P*-value < 0·001), but not lysine deficiency (*P*-value = 0·091), was able to increase the weight of the spleen, while the opposite results stood for the adipose tissue where lysine deficiency (*P*-value < 0·001) but not LPS (*P*-value = 0·085) resulted in a decrease in fat accumulation. Interestingly, lysine deficiency decreased liver weight (*P*-value = 0·012) while LPS increased it (*P*-value < 0·001), irrespective of its fat content (*P*-value = 0·067).

**Fig. 3.**
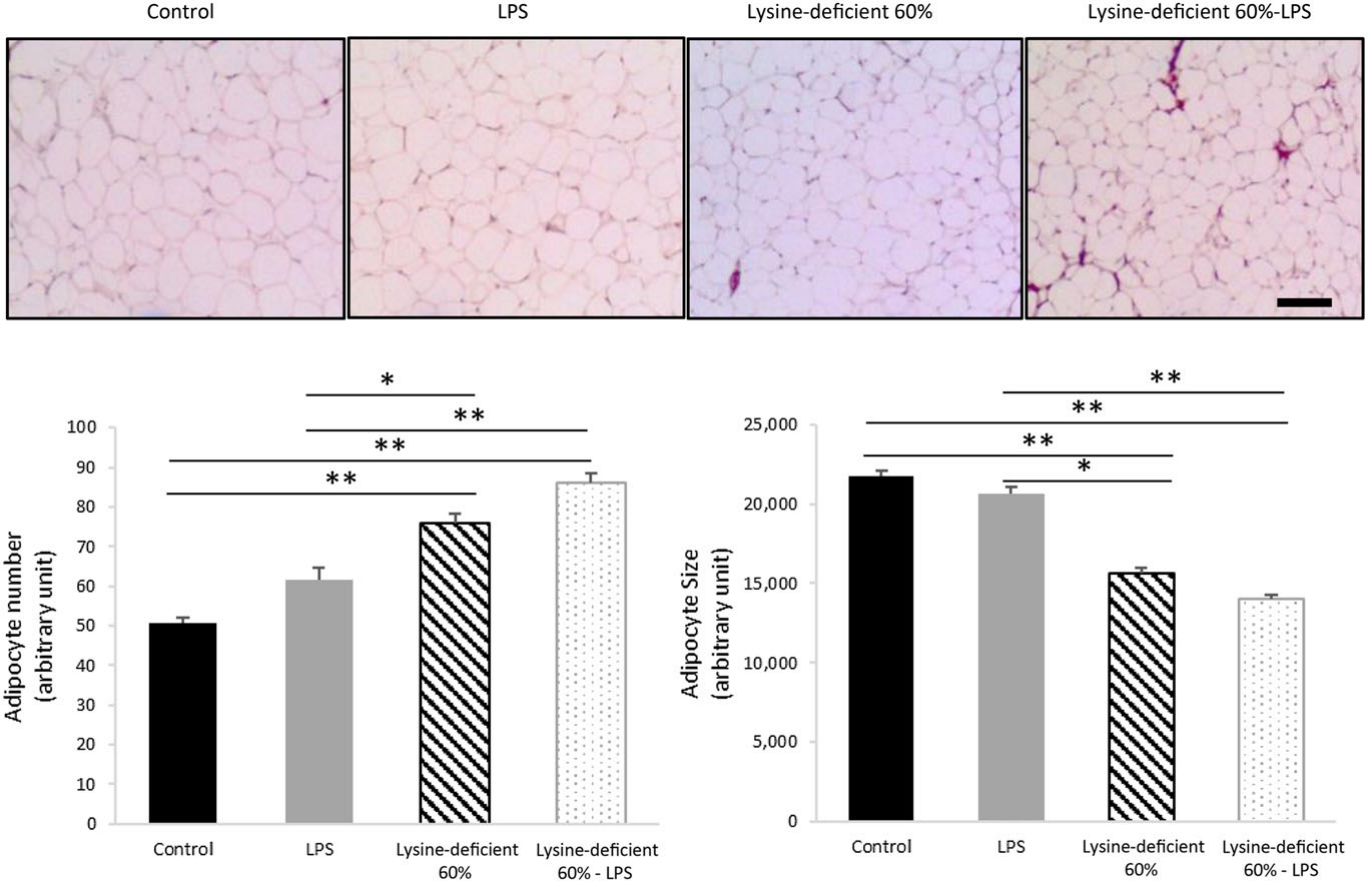
Hematoxylin and eosin (H&E) staining was done on sections of the epidydimal adipose tissue of rats fed a control diet or a 60 % lysine-deficient diet in the presence or absence of LPS challenge (*n* = 5 per group). Photos were taken at 4× magnification under a light microscope. Scale bar represents 100 µm. The size of adipocytes was estimated using ImageJ software, and quantification was done manually for a set area in all slides. Data are expressed as mean ± sem and analysed by one-way ANOVA to detect the difference between groups, followed by Fisher’s least significant difference for *post hoc* multiple comparisons. Statistical significance was set at *P*-value < 0·05. * refers to *P*-value < 0·05. ** refers to *P*-value < 0·01. LPS, lipopolysaccharide.

Adipocyte number was different between groups (*P*-value = 0·004), with an increase in the count with both lysine deficiency and LPS. Interestingly, the size of the adipocytes was smaller in both lysine-deficient groups (*P*-value < 0·001) (Fig. 3).

No morphological changes were seen at the liver level, and mononuclear cell infiltration was not detected either at lobules or at the portal tracts (online Supplementary Fig. S2).

### Blood measurements

Lysine deficiency significantly diminished plasma levels of TAG, cholesterol and HDL (Table 1). Interestingly, rats with LPS challenge had higher lipoprotein levels. LDL-cholesterol was found to increase with lysine deficiency, yet the rise was not statistically significant due to the large variation in the samples. A remarkable surge in blood chloride, but not Na or lactate, was also associated with lysine deficiency (Table 1).

**Table 1.** Fasting plasma lipid profile and pH-medicated parameters of rats at end point

	Control		LPS		Lysine-deficient 60 %		Lysine-deficient 60 %-LPS		ANOVA *P*-value
	Mean	sd	Mean	sd	Mean	sd	Mean	sd	
Lipid profile									
TAG (mg/dl)	82·0	14·6^a^	112·0	44·5^b^	40·6	6·9^c^	38·7	6·2^c^	<0·001
Total cholesterol (mg/dl)	91·3	6·6^ac^	96·8	16·6^a^	76·5	12·8^b^	82·0	6·1^bc^	0·009
HDL (mg/dl)	69·7	4·5^a^	71·5	11·3^a^	54·4	6·7^b^	65·0	5·2^a^	<0·001
LDL (mg/dl)	5·3	4·1	8·0	5·4	11·0	4·8	8·8	2·0	0·109
pH-mediated parameters									
Na (mmol/l)	136·6	1·2	136·4	0·8	135·6	1·3	135·7	1·6	0·302
Chloride (mmol/l)	92·3	1·2^a^	91·6	0·8^a^	97·8	2·4^b^	96·4	1·6^b^	<0·001
Lactate (mmol/l)	8·7	2·1	10·1	1·7	10·1	0·9	9·5	2·5	0·409

Data are expressed as the mean ± sd and analysed by one-way ANOVA to detect the difference between groups, followed by Fisher’s least significant difference for *post hoc* multiple comparisons (*n* = 8 per group). Means with the same letter are not significantly different from each other (*P*-value > 0·05).

Statistical significance was set at *P*-value < 0·05.

An intraperitoneal glucose tolerance tests was performed on four rats per group (online Supplementary Fig. S3A and S3B). Results from the general linear model showed that lysine deficiency diminished a normal glycaemic response at all time points (*P*-value = 0·024) (online Supplementary Fig. S3A); however, the decrease in LPS-challenged rats was not different (*P*-value = 0·321). Despite the clear trend in the glycaemic response, the AUC was comparable among groups (*P*-value = 0·099) (online Supplementary Fig. S3B). Similarly, no differences were detected for fasting plasma glucose (*P*-value = 0·389), insulin (*P*-value = 0·595), glucagon-like peptide-1 (*P*-value = 0·774) and HOMA-IR (*P*-value = 0·645) (online Supplementary Figures S3C–S3F).

Plasma levels of free amino acids in all rats were also assessed. As shown in Table 2, essential amino acids, along with glycine, serine and tyrosine, were different with lysine deficiency. It is worth noting that lysine deficiency decreased most amino acids except for histidine, threonine and serine, which were shown to rise. The general linear model analysis affirmed that the existent variation in amino acid concentration was exclusively related to the content of lysine in the diet, and no direct effect of LPS (or interaction) was detected. The sum of essential amino acids, non-essential amino acids and the total was similar among groups.

**Table 2.** Plasma amino acid levels in rats at end point

Amino acids (µmoles/l)	Control		LPS		Lysine-deficient 60 %		Lysine-deficient 60 %-LPS		ANOVA *P*-value
	Mean	sd	Mean	sd	Mean	sd	Mean	sd	
Essential amino acids									
Histidine	86·9	14·0^a^	88·4	20·8^a^	127·1	30·6^b^	129·3	21·9^b^	0·001
Isoleucine	109·6	20·1^a^	116·6	22·9^a^	85·5	19·1^b^	79·2	16·6^b^	0·003
Leucine	197·1	33·1^ab^	207·5	29·9^a^	168·0	29·9^bc^	152·8	21·4^c^	0·006
Lysine	452·0	131·7^a^	472·4	47·1^a^	220·6	56·8^b^	190·5	27·8^b^	<0·001
Methionine	55·5	15·8^a^	56·8	7·9^a^	34·8	13·6^b^	31·0	4·2^b^	<0·001
Phenylalanine	88·4	12·2^a^	90·5	10·5^a^	60·4	10·1^b^	51·8	7·1^b^	<0·001
Threonine	243·6	42·7^a^	241·5	48·3^a^	653·8	314·8^b^	587·2	171·4^b^	<0·001
Tryptophan	78·9	21·6^a^	77·5	14·0^a^	40·6	11·6^b^	46·8	12·4^b^	<0·001
Valine	211·8	29·1^a^	209·9	26·4^a^	161·6	29·6^b^	146·7	17·9^b^	<0·001
Total	1523·8	258·1	1561·0	163·8	1552·4	394·0	1415·3	195·1	0·759
Non-essential amino acids									
Alanine	748·1	115·2	784·6	108·8	833·6	169·6	781·3	175·9	0·696
Arginine	88·4	55·5	98·1	67·0	88·9	36·9	85·5	29·8	0·967
Asparagine	59·3	18·5	59·9	15·7	49·9	11·7	46·4	8·0	0·277
Citrulline	103·8	14·1	102·0	9·1	101·9	13·1	90·2	13·7	0·217
Glutamic acid	346·4	109·6	403·4	95·2	366·8	114·2	292·0	80·0	0·261
Glutamine	766·4	147·0	766·4	92·9	917·4	176·2	885·5	134·5	0·090
Glycine	362·9	67·7^ab^	435·8	91·4^b^	318·0	61·1^a^	328·0	12·9^ab^	0·011
Ornithine	144·9	83·0	137·6	82·0	104·9	60·9	82·0	38·6	0·326
Serine	405·6	40·4^a^	399·5	64·3^a^	586·5	128·9^b^	563·7	82·0^b^	<0·001
Taurine	260·0	76·8	300·4	59·1	292·8	82·8	276·7	29·6	0·646
Tyrosine	85·5	36·8^a^	87·4	12·1^a^	65·5	15·7^ab^	40·5	15·1^b^	0·002
Total	3371·1	362·7	3575·0	351·1	3719·8	512·0	3409·3	401·9	0·343
Overall total	4895·9	579·4	5137·0	457·9	5273·1	857·8	4825·7	507·9	0·504

Data are expressed as the mean ± sd and analysed by one-way ANOVA to detect the difference between groups, followed by Fisher’s least significant difference for *post hoc* multiple comparisons (*n* = 8 per group). Means with the same letter are not significantly different (*P*-value > 0·05). Statistical significance was set at *P*-value < 0·05.

### Albumin and inflammatory markers

**Fig. 4.**
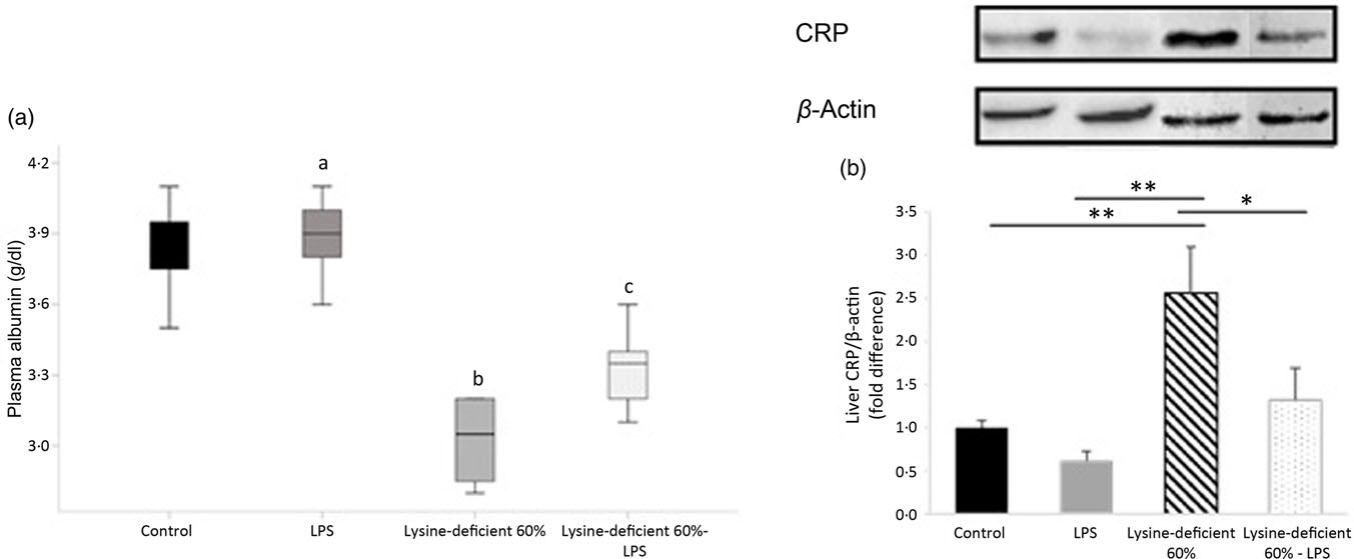
Fasting plasma albumin and CRP expression in the liver of rats fed a control diet or a 60 % lysine-deficient diet in the presence or absence of LPS challenge (*n* = 8 per group). Protein bands detected by western blots were quantified with Image J software and normalised against *β*-actin. Plasma levels are expressed as median (the bar inside the box), the interquartile range (the box length), the minimum value (the lower bar cap) and the maximum value (the upper bar cap). The western blot quantification is expressed as mean ± sem. Statistical significance was shown by one-way ANOVA to detect the difference between groups, followed by Fisher’s least significant difference for *post hoc* multiple comparisons. Means with the same letter are not significantly different (*P*-value > 0·05). Statistical significance was set at *P*-value < 0·05. * refers to *P*-value < 0·05. ** refers to *P*-value < 0·01. CRP, C-reactive protein; LPS, lipopolysaccharide.

Both plasma albumin levels (*P*-value < 0·001) and hepatic protein expression of CRP (*P*-value = 0·006) were different between the groups (Fig. 4). Lysine deficiency decreased the levels of albumin (*P*-value < 0·001), in contrast to the increase in hepatic protein expression of CRP (*P*-value = 0·004). Hepatic CRP expression increased by about 2·5-fold in the lysine-deficient group compared with the control group. On the other hand, a decrease in hepatic protein expression of CRP was associated with LPS administration.

**Fig. 5.**
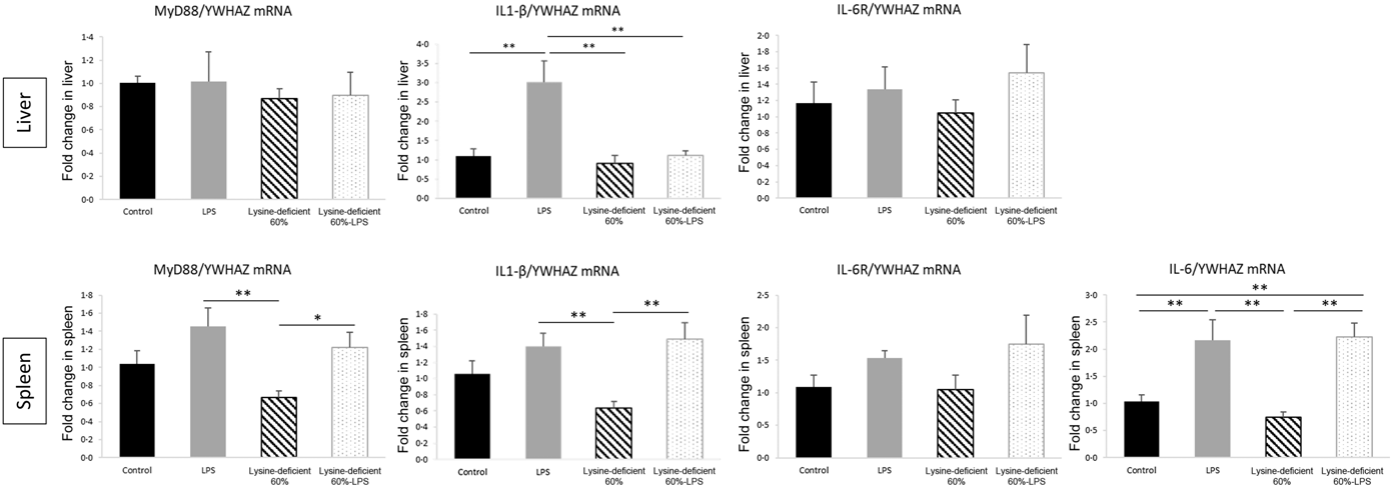
qRTPCR results showing the relative mRNA expression of MyD88, IL-1*β*, IL-6 and IL-6R in the liver and the spleen of rats fed a control diet or a 60 % lysine-deficient diet in the presence or absence of LPS challenge (*n* = 5 per group). Data are expressed as mean ± sem and analysed by one-way ANOVA to detect the difference between groups, followed by Fisher’s least significant difference for *post hoc* multiple comparisons. Statistical significance was set at *P*-value < 0·05. * refers to *P*-value < 0·05. ** refers to *P*-value < 0·01. LPS, lipopolysaccharide.

Adjusted gene expression to housekeeping gene YWHAZ was assessed relative to the control group. In the liver, myeloid differentiation primary response 88 (MyD88) (*P*-value = 0·897) and IL-6 receptor (IL-6R) (*P*-value = 0·624) mRNA expressions were not different among groups. Nonetheless, unlike all other groups, the LPS group expressed a high IL-1*β* (*P*-value = 0·001) (Fig. 5).

In the spleen, MyD88 was different among groups (*P*-value = 0·015) with an increment with LPS administration (*P*-value = 0·006). A similar pattern was described for IL-1*β* (*P*-value = 0·011) and IL-6 (*P*-value = 0·001). Despite the clear trend, IL-6R increase with LPS did not reach statistical significance (*P*-value = 0·250) (Fig. 5).

### Behavioural tests

**Fig. 6.**
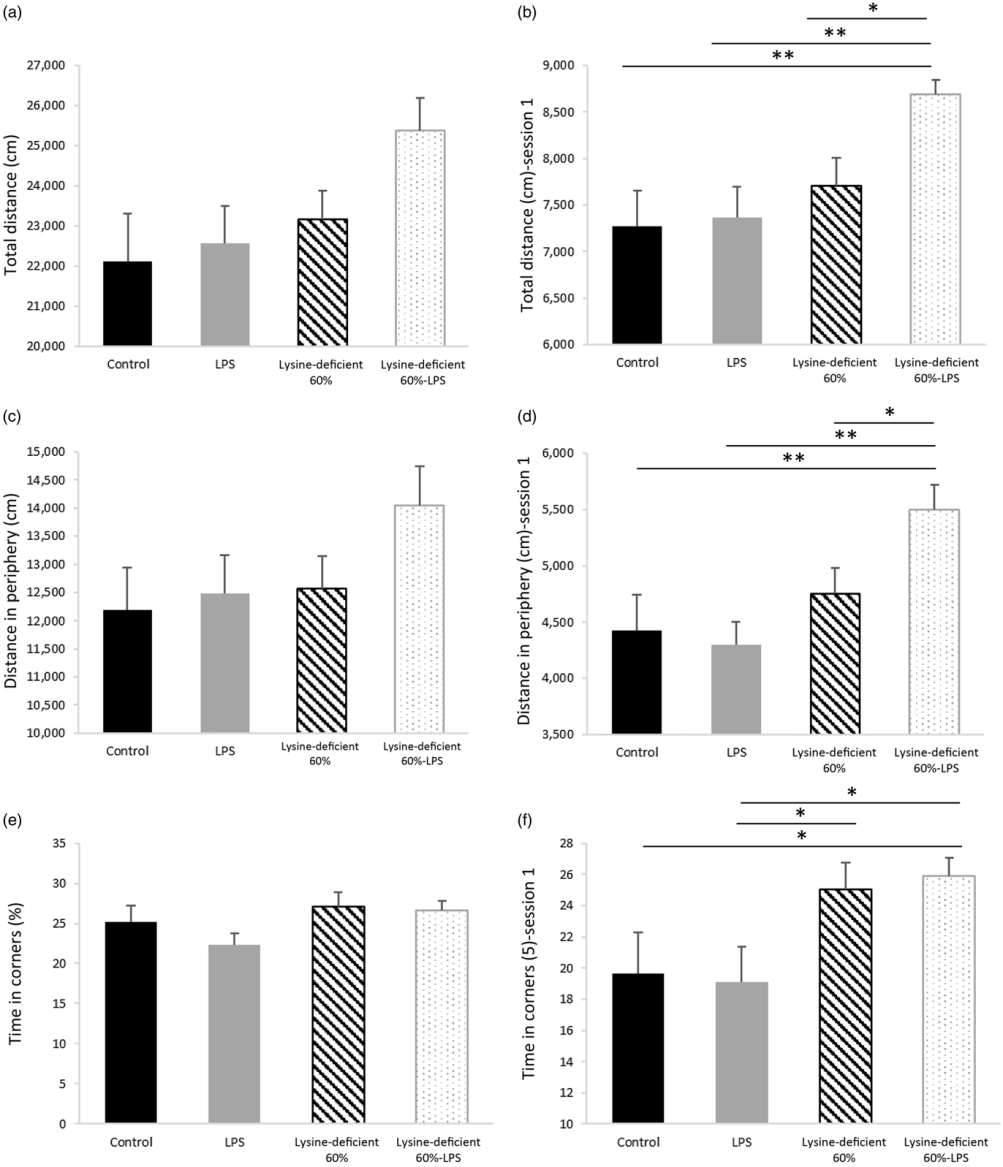
(a) Total distance travelled during the three consecutive sessions combined, (b) total distance travelled in session 1, (c) distance travelled in the peripheral area during the three consecutive sessions combined, (d) distance travelled in the peripheral area in session 1, (e) percent time spent in the corners during the three consecutive sessions combined, and (f) percent time spent in the corners in session 1 in rats fed a control diet or a 60 % lysine-deficient diet in the presence or absence of LPS challenge (*n*=8 per group). Data are expressed as mean ± sem and analysed by one-way ANOVA to detect the difference between groups, followed by Fisher’s least significant difference for *post hoc* multiple comparisons. Statistical significance was set at *P*-value < 0·05. * refers to *P*-value < 0·05. ** refers to *P*-value < 0·01. LPS, lipopolysaccharide.

The pain assessment was performed a week before euthanasia using the Hargreaves test and showed no difference among the groups (*P*-value = 0·501) (online Supplementary Fig. S4). In addition, the open-field test data in Fig. 6 illustrate the total distance travelled (Fig. 6(a) and (b)), the distance travelled in the peripheral areas (Fig. 6(c) and (d)) and the time spent in corners (Fig. 6(e) and (f)). Figure 6(a), (c) and (e) summarise the total indicators of all three sessions combined and failed to show any statistical difference between groups (*P*-value > 0·05). However, Fig. 6(b), (d) and (f) demonstrate the same parameters in session 1 and display discrepancies in performance with an increase in the total and peripheral distance travelled and the time spent in corners in the lysine deficiency groups, but this increment was more pronounced in the presence of LPS. While ANOVA shows a significant exacerbation of the results among the lysine-deficient 60 %-LPS group, the general linear model reveals that the three parameters resulted from lysine deficiency and that LPS administration did not have a major effect on the behavioural assessment. Representative trajectories are illustrated in Supplementary Fig. S5.

## Discussion

### Body measurements and energy balance

The ingestion of a lysine-deficient diet was found to halt growth and development, which was in line with the literature^(14,31,32)^. In fact, lysine operates as a building block for protein synthesis as it serves as an important precursor of the signalling pathway for the mammalian target of rapamycin (mTOR)^(33)^. In addition, lysine deficiency was found to increase the fractional rate of proteolysis, leading to more loss in muscle mass^(34)^.

While lysine in diet contributed to the main anthropometric differences, LPS has also conferred a minor yet significant effect. It has been shown that in the case of any immune response (triggered by LPS), amino acid repartitioning takes place, favouring the activation of the immune system and compromising protein synthesis related to growth^(35)^. In addition, pro-inflammatory cytokines can detrimentally affect the thyroid gland and consequently blunt growth and development^(36)^. In line, immune reactions are known to increase lipolysis, resulting in a decrease in fat mass and an upsurge of free fatty acid in the circulation. This phenomenon can happen through the binding of LPS (via its lipid A moiety) to its receptor toll-like receptor 4 and stimulating its downstream inflammatory pathway^(37)^. Another potential lipolytic route is through the inositol-requiring protein 1, a component of the endoplasmic reticulum stress whose activation is insulin-independent^(38)^. The lipolytic cycles, feeding the circulation with free fatty acids, further stimulate the inflammatory outcomes through toll-like receptor 4^(39)^, dragging the system into a vicious cycle. It is worth noting that despite the change in body fat mass, the fat percentage among groups at baseline and end point did not change.

Interestingly, the difference in body weight and composition was not related to the total energy intake (which was similar) but to energy efficiency. In fact, the effect of the amino acid restriction on energy intake depends on the magnitude of the deficiency. While a 75 % lysine deficiency increased feed intake and decreased body weight, Kim *et al.* reported no difference in both parameters when deficiency is as low as 50 %^(40)^. In this work, the energy expended was superior to the energy used for growth and development, and this can be explained as follows. First, rats that consumed lysine-deficient diets were hyperactive, and this behaviour was exacerbated with LPS administration. Thus, more energy was consumed to support their increased physical activity. Second, when sufficient amounts of essential amino acids are unavailable for protein synthesis, more amino acids will remain in circulation. As a protective mechanism against amino acid accumulation, the body starts oxidising more amino acids which results in an increase in energy expenditure and a general catabolic state, hindering fat and lean mass accumulation. Consequently, the amount of energy used for growth and weight gain would decrease. The increase in energy expenditure and amino acid oxidation following a low-quality-protein diet is not restricted to lysine deficiency only^(14,41,42)^.

The size of the organs of the rats confirmed the body composition findings. Spleens were enlarged in the presence of LPS, showing an active immune status, which was in line with other studies^(43,44)^. The similarity in fat percentage in dry livers between the groups implies that the increase in the liver size with LPS is not attributed to an ectopic lipid accumulation but to the liver involvement in the body’s immune response^(45)^. Nevertheless, the duration of exposure (4 weeks), dose of LPS (150 μg/kg/week) and time of euthanasia (24-h post last LPS injection) were insufficient to show any morphological changes or mononuclear cell infiltration in the livers. However, it is confirmed that both LPS administration and lysine deficiency have caused functional alterations.

A decrease in body fat gain paralleled the decline in epididymal adipose tissue weight, which was attributed to the lack of lysine. The adipose tissue dynamics also differed among the groups, and this was seen at a microscopic level. The adipocytes were more numerous and smaller in size in the lysine-deficient diet, and these alterations pose serious cardiovascular implications^(46)^, believing they are associated with higher levels of circulating free fatty acids.

### Blood measurements

An interesting characteristic of the lysine-deficient diet was the drop in lipid profile (except for the LDL) that is widely reported in the community among malnourished populations^(47,48)^. Remarkably, lysine is largely needed for the synthesis of lipoproteins^(49)^. Also, LPS worked differently with the diets manipulating lipid profiles; it increased the concentrations in combination with the control but not lysine-deficient diet. In a well-nourished status, high levels of NEFA induced by LPS^(50)^ are esterified to plasma TAG, a mechanism to reduce the cytotoxic effect of NEFA.

Another remarkable alteration caused by lysine deficiency was the increase in chloride levels. When the pH of the medium decreases, the total unbound NEFA concentration increases, exacerbating the health status^(51)^.

Protein and amino acid metabolisms change drastically during malnutrition. Due to the low lysine content in diet, plasma lysine dropped to a similar extent (50 to 60 % decrease). In addition, the deficient rats witnessed a fall in most other amino acids which is considered a hallmark associated with malnutrition^(52)^. It is worth mentioning that, unlike the decrease in levels seen with the majority of amino acids, histidine and serine increased by about 50 % and threonine by 2·5-fold with the deficient diet. The increase in threonine levels (and at a lower extent in histidine and serine) in lysine deficiency was previously reported in the literature^(53–57)^ and could be explained by a hindered utilisation and/or oxidation of these amino acids.

In addition, amino acid concentration (essential, non-essential and total) was comparable between groups, showing how the body compensates for the drop in some amino acids with the increase in others. In harmony with the hypothesis that affirms a relationship between serum amino acid concentration and appetite^(58)^, similar levels of circulating amino acids might have contributed to comparable feed and energy intake. The 60 % lysine deficiency in this work was low to cause the classical malnutrition manifestation but not low enough to alter total amino acid concentration and drop food intake.

### Albumin and inflammatory markers

This study proved an inverse relationship between plasma albumin concentration and liver CRP expression. Some observations (secondary data in most cases) have acknowledged an inverse relationship between albumin and CRP levels in particular cases. Mosli & Mosli^(59)^ showed an inverse association between albuminemia and obesity, which independently elevates CRP^(60–63)^. Patients with celiac disease also present alterations in their blood parameters with low levels of albumin^(64)^ and high levels of CRP^(65)^. Nonetheless, some researchers suggested a CRP (or high-sensitivity CRP) to albumin or prealbumin ratio to identify the activity of some inflammatory diseases^(66–68)^.

Interestingly, the most dominant essential amino acid in the sequence of albumin is lysine^(69)^. Consequently, by diminishing lysine supply through a deficient diet, not enough lysine remains available to produce this big plasma protein. This finding has great clinical implications seeing the major role albumin plays in health and disease^(70–72)^. This work also shows that LPS administration was correlated with an increase in albumin levels. This result corresponds to the role of albumin as a carrier protein and an immune modulator. In fact, LPS can bind to albumin and form a complex that is much less effective in activating toll-like receptor 4 than is LPS-Lipid A^(73)^.

Remarkably, an exact opposite pattern was reported for liver CRP. Lysine deficiency alone increased liver CRP expression by about 2·5-fold, independently from IL-6 and IL-1*β*, the main precursors of CRP^(74,75)^. In fact, albumin is three times the size of CRP (585 amino acids for albumin^(76)^ *v*. 206 for CRP^(77)^) with a similar amino acid profile; leucine and lysine are the two most abundant essential amino acids in the CRP composition^(78)^. In brief, under lysine deficiency, the body seems to prioritise the production of small transport proteins requiring less lysine (CRP) at the expense of large proteins (albumin). Therefore, this fact explains the reduction of CRP levels when lysine and leucine were supplemented^(79)^ and albumin regained its initial transport role.

The livers and spleens did not display a similar gene expression profile. MyD88, a downstream target of the toll-like receptor 4, and IL-6R mRNA expressions were similar among groups, while IL-6 was not expressed in the liver. The only difference at the hepatic level was the expression of IL-1*β* for the LPS group. It is worth mentioning that among deficient rats, the expression of IL-1*β* was similar to the unchallenged group. This clearly states that the liver does not mount a normal reaction to antigens in case of deficiency but prioritises internal regulations. Then again, the spleen exhibited more discrepancies between groups, where MyD88, IL-1*β* and IL-6 gene expressions all increased with LPS. In fact, the liver and spleen convey pro-inflammatory cytokine expression at different rates, while hepatic reactions are faster and shorter, the splenic ones are slower and more consistent^(80)^.

Previous work in the literature has come in support of our findings. A study on pigs showed that supplementation with functional amino acids improved the system’s defence and immune function^(81)^. Inline, medical records from hospitalised patients with systemic inflammation (CRP > 0·5 mg/dl) proved that when patients were supplemented with essential amino acids, CRP dropped while the immune cells in the circulation increased under inflammatory and infectious conditions^(79)^.

### Behavioural tests

An assessment of pain sensation in rats showed no difference among groups, affirming that the results presented in the open-field test were not caused by painful motions. LPS dose and frequency in this work were not enough to provoke pain, unlike what is commonly reported about LPS-inducing hyperalgesia^(82–84)^. Nonetheless, Yirmiya *et al.* demonstrated that the administration of LPS (200 μg/kg, which is four times the dose in this study) increased pain sensitivity 2 h after injection but only lasted for about 30 h^(85)^.

Lysine deficiency increased the total distance travelled by the rats and the distance in the periphery, suggesting hyperactive behaviour, which was exacerbated by LPS administration. No effect of LPS was seen when the diet was complete, which suggests a protective role of lysine at a central level against the harm that LPS causes.

Additionally, rats on deficient diets spent more time in the corners, which indicates an anxiety-like behaviour. This result seen in session 1 and lost in the subsequent sessions might be attributed to the habituation of the rats, their learning ability and memory that were maintained. It is worth noting that lysine acts as a glutamate precursor in the mammalian central nervous system^(86)^, and a selective reduction of glutamate in the ventral hippocampus is associated with anxiety-like characteristics in rats^(87)^. Also, lysine can work as an antagonist of serotonin receptors in the brain and provide an antidepressant effect, similar to the findings of Cremers *et al.* ^(88)^ In a study on guinea pigs, lysine inhibited the binding of serotonin to its receptor 4 (5-HT4), reducing anxiety-related diarrhoea^(89)^. Moreover, as a carrier protein, albumin binds to amino acids for their transport. Any decrease in plasma albumin level (by a decrease in production) or availability (by an increase in free fatty acids) would be expected to increase the availability of free tryptophan^(90)^ that competes with other large neutral amino acids on their transporters at the blood–brain barrier. Consequently, the imbalance in amino acids in the brain alters the production of the varied neurotransmitters.

We acknowledge the importance of conducting the same study on female rats considering the discrepancies in serum albumin concentrations between sexes, especially at a young age^(91)^, and knowing that females have a compensatory mechanism that alleviates the response to endotoxins^(92)^. These sex differences highlight a main limitation of our study. Another limitation would be the lack of prealbumin assessment that would support the findings and hypothesis, given its high lysine content^(93)^.

In conclusion, this study confirmed that lysine deficiency hinders the production of albumin and stimulates hepatic CRP expression. This status is accompanied by an increase in anxiety-like behaviour, a blunted growth and an alteration in some inflammatory markers’ expressions. Moreover, with LPS insult, lysine deficiency exacerbated hyperactivity, blood and tissue parameters, and modulated the immune response. This project adds to the recent advancements and understanding of the interplay between nutrition and inflammation, shedding light on amino acids and protein quality. It remains to be understood whether other forms of low-quality protein would exhibit similar effects. We believe that the increment in non-communicable disease among the low socio-economic status populations can be, at least partially, blamed on low lysine availability in their diets.

## Supplementary material

For supplementary material accompanying this paper visit https://doi.org/10.1017/S0007114522004068. click here to view supplementary material
