# Supplementary material for: Low-quality protein modulates inflammatory markers and the response to lipopolysaccharide insult: the case of lysine
Source: Br J Nutr. 2023 Jan 4;130(6):944–57. doi: 10.1017/S0007114522004068 (PMC10442798; doi:10.1017/S0007114522004068)
Supplement: Supplementary file 1 [file S0007114522004068sup001.docx]

**Table S1. Diet composition of the experimental diets**

| **Ingredients (g/kg)** | **Control Diet** | **60% Lysine**  **Deficient Diet** |
| --- | --- | --- |
| Wheat gluten*^‖^ | 188.2 | 196.1 |
| Cornstarch | 388.1 | 387.6 |
| Sucrose | 232 | 232.1 |
| Corn oil | 70 | 70 |
| Cellulose* | 50 | 50 |
| Mineral mix*^₶^ | 35 | 35 |
| Vitamin mix*^£^ | 10 | 10 |
| Choline Bitartrate | 2.5 | 2.5 |
| L-Methionine | 3 | 3 |
| L-Threonine | 0.5 | 0.5 |
| Potassium phosphate*^¥^ | 13.2 | 13.2 |
| L-Lysine HCl*^¶^ | 7.5 | - |

*Obtained from Dyets Inc., Bethlehem, Pennsylvania, USA.

^‖^The diets contained 10% protein coming exclusively from wheat gluten (76% protein).

^₶^"Phosphorus-free" mineral mix (AIN-93G mineral mix phosphorus-free, used as 35 g/kg of diet).

^£^Vitamin mix (AIN-93VX vitamin mix, used as 10 g/kg of diet).

^¥^Potassium phosphate KH2PO4, molecular weight 136 g/mol, of which phosphorus 31 g/mol.

^¶^L-Lysine HCl, molecular weight 182 g/mol, of which lysine 146 g/mol.

**Table S2. The nutrition facts of the experimental diets**

| **Nutrients** | **Control Diet** | **60% Lysine-**  **Deficient Diet** |
| --- | --- | --- |
| Protein* (%) | 15.4 | 15.4 |
| Carbohydrates* (%) | 67.9 | 67.9 |
| Fat* (%) | 15.9 | 15.9 |
| Total energy (kJ/kg) | 16,418 | 16,426 |
| Lysine (g/kg) | 9.8 | 3.8 |

*values represent a percentage of total energy.

The nutrient content of diets was estimated based on the nutrition information on the label of the products.

**Table S3. Primer sequences used in qRT-PCR**

| Rattus norvegicus gene | Sequence (5'- > 3') | Product size (pb) | Melting temperature |
| --- | --- | --- | --- |
| YWHAZ | F: CCCACTCCGGACACAGAATA  R: TGTCATCGTATCGCTCTGCC | 91 | 82 |
| MyD88 | F: GAAATACATACGCAACCAGCAGAAA  R: CAGATGAAGGCGTCGAAAAGC | 156 | 86 |
| IL-6 | F: ACAAGTCCGGAGAGGAGACT  R: ACAGTGCATCATCGCTGTTC | 167 | 80 |
| IL-6R | F: AGCAGGCAATGCTACCATTCAC  R: GTCGGTATCGAAGCTCGAATTG | 611 | 86 |
| IL-1β | F: AGGCTGACAGACCCCAAAAG  R: GGTCGTCATCATCCCACGAG | 264 | 85 |

Primers were brought from Macrogen (South Korea).


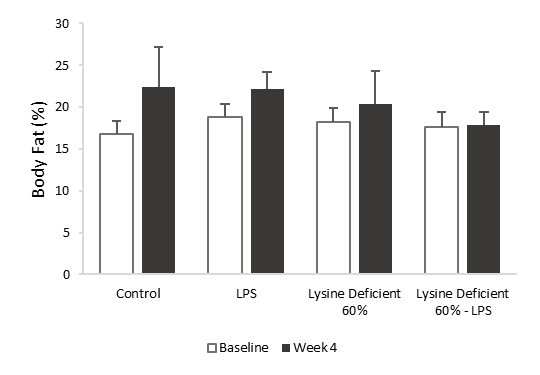


**Figure S1. Percentage body fat**


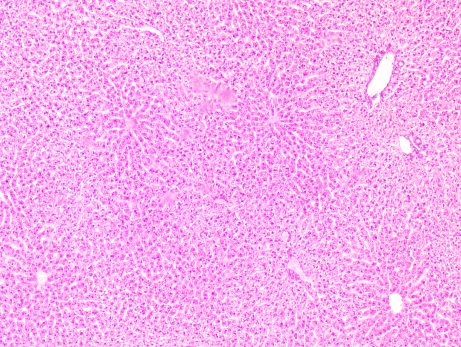

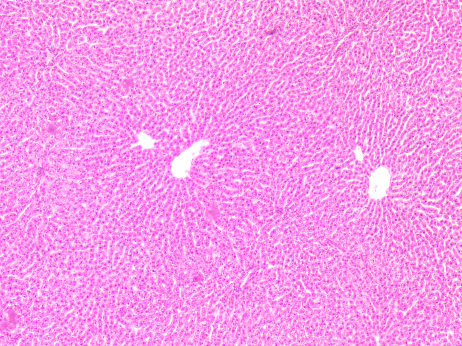


Control

LPS


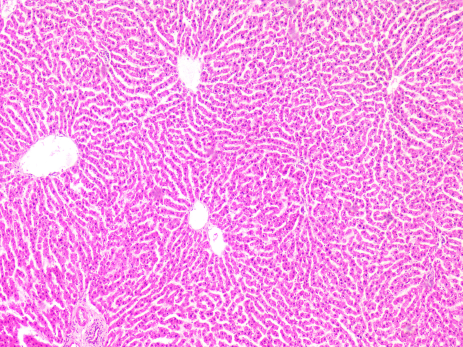

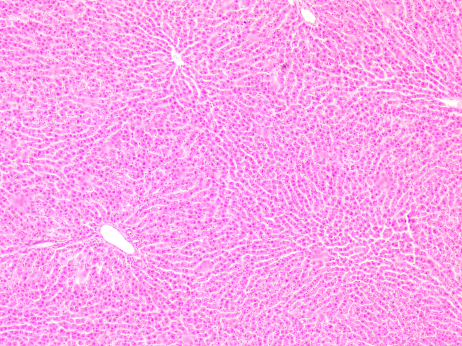


Lysine Deficient 60%

Lysine Deficient 60% - LPS

**Figure S2. Representative H&E staining of liver sections from the different groups of rats**

Histopathological appearance of liver sections stained with hematoxylin–eosin (H&E) at ×4 magnification power rats fed a control diet or a 60% lysine deficient diet in the presence or absence of LPS challenge (n = 5 per group). Scale bar represents 100 µm.


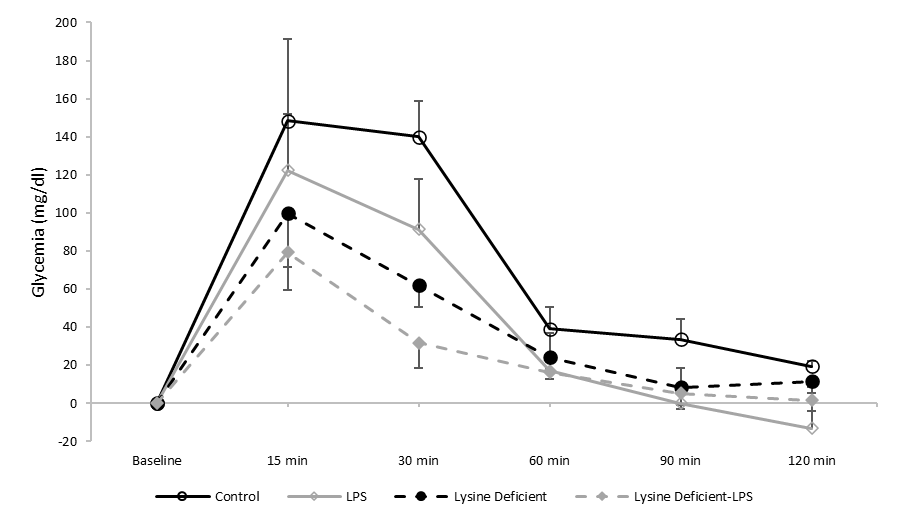


**A**


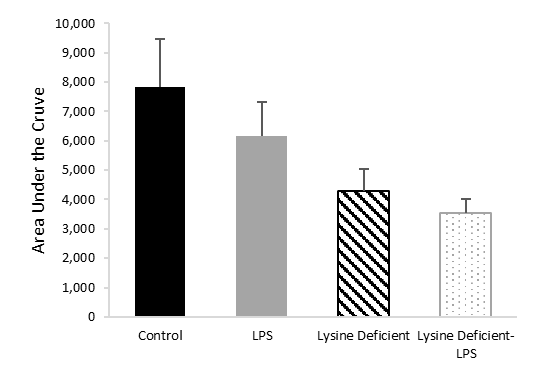


**B**

**F**


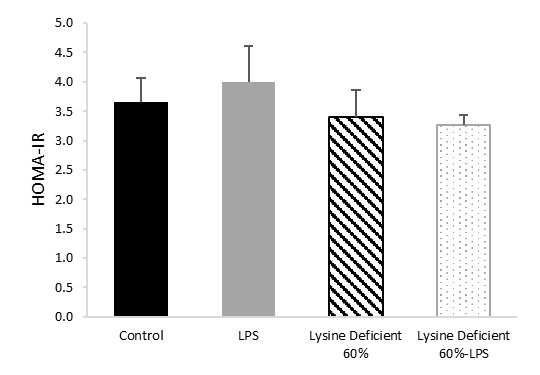


**E**


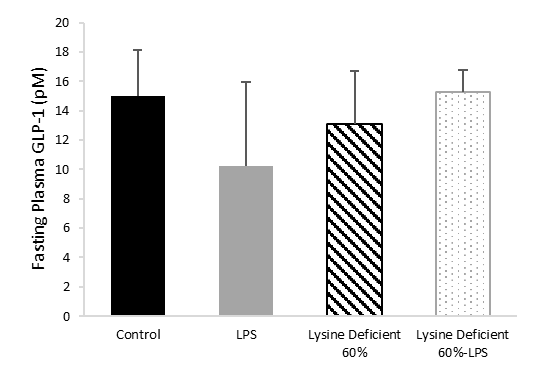


**D**


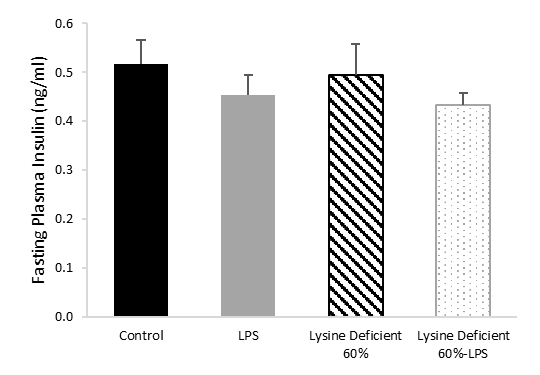


**C**


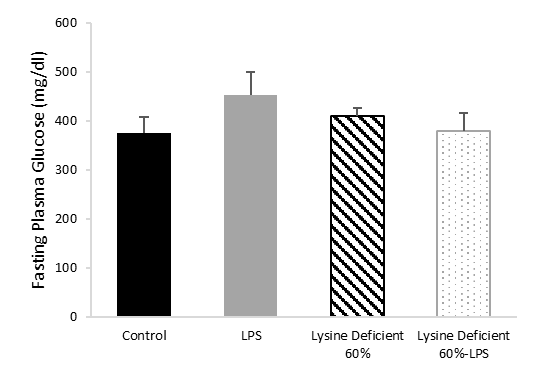


**Figure S3. Glycaemic profile of rats**

(A) Intraperitoneal glucose tolerance test (IPGTT) (n=4), (B) Area under the curve of the IPGTT, (C) Fasting plasma glucose (n=8), (D) Fasting plasma insulin (n=8), (E) Fasting plasma GLP-1 (n=8), and (F) HOMA-IR index (n=8) were measured in rats fed a control diet or a 60% lysine deficient diet in the presence or absence of LPS challenge.

Data are expressed as mean±SEM and analysed by one-way ANOVA to detect the difference between groups, followed by Fisher's least significant difference (LSD) for post hoc multiple comparisons.

Statistical significance was set at p-value<0.05.


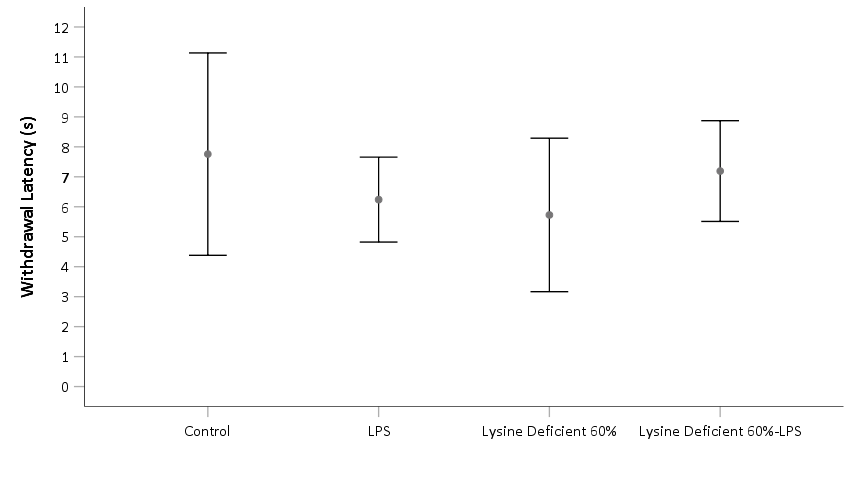


**Figure S4. Thermal pain sensation in rats**

This heat stimulus was applied thrice per rat, separated by an interval of 5 minutes. The average of the two closest values was reported. Data are expressed as mean±SEM and analysed by one-way ANOVA to detect the difference between groups, followed by Fisher's least significant difference (LSD) for post hoc multiple comparisons.

Statistical significance was set at p-value<0.05.


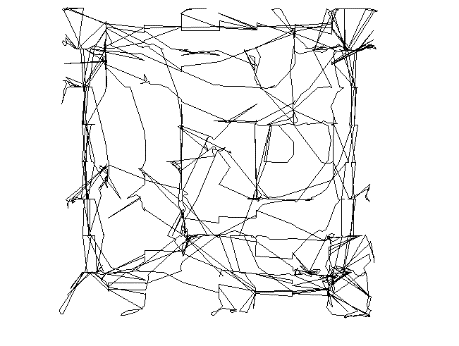

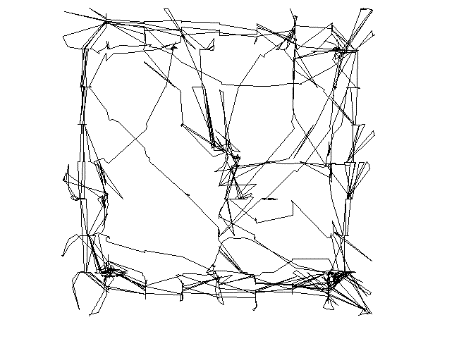

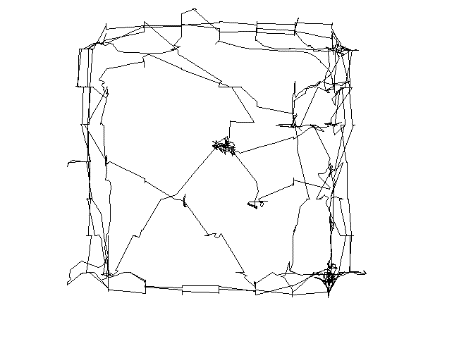

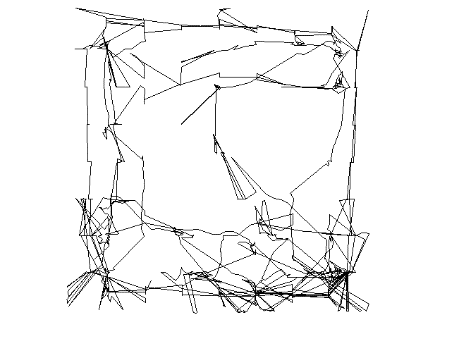


**Control**

**LPS**

**Lysine Deficient 60%**

**Lysine Deficient 60%-LPS**

**Figure S5. Sample trajectory of the group during session 1 of the Open Field Test in rats**

The trajectories were recorded for 5 minutes via a video camera connected to the tracking software Smart.
